# Bioelectrical impedance analysis in the health field: a still scientifically fragile tool for body composition assessment

**DOI:** 10.1590/1806-9282.20250655

**Published:** 2025-09-19

**Authors:** André Pontes-Silva, Daria Andrusik, Yury Zharikov

**Affiliations:** 1 Universidade Federal de São Carlos, Department of Physical Therapy, Postgraduate Program in Physical Therapy – São Carlos (SP), Brazil.; 2 Federal State Budgetary Educational Institution of Higher Education, Russian University of Medicine Ministry of Health of the Russian Federation – Moscow, Russia.; 3 FSAEI HE I.M. Sechenov First MSMU of MOH of Russia (Sechenovskiy University) – Moscow, Russia.

Dear Editor,

The paper entitled "Bioelectrical impedance analysis and skinfold thickness for the estimation of body fat: A population-based study in southern Brazil," published in the Revista da Associacão Médica Brasileira (2025), compared bioelectrical impedance analysis (BIA) and skinfold measurements for estimating body fat in adults. Arlindo Sousa et al. concluded that the two methods (BIA and skinfold) showed good agreement between the mean values of body fat percentage^1^. This is a wonderful study^1^. I sincerely congratulate the authors on their excellent work^1^. My scientific critique focuses exclusively on highlighting the scientifically fragile nature of the BIA for body composition assessment. The use of BIA as a bedside method has increased because the equipment is portable and safe, the procedure is simple and "non-invasive," and the results are reproducible and rapid^2^. More recently, segmental BIA has been developed to overcome inconsistencies between trunk resistance and body mass^3^. However, it is still a scientifically fragile tool for body composition assessment^4^.

In addition, BIA measurements must be standardized to obtain reproducible body composition: reported mean coefficients of variation for within-day resistance measurements are ≈1–2%; daily or weekly intra-individual variability is slightly greater, ranging from ≈2 to 3.5%; daily coefficients of variation increase for frequencies below 50 kHz^3,4^. Overall reproducibility/precision is 2.7–4.0%. Prediction errors have been estimated to be 3–8% for total body water and 3.5–6% for fat-free mass (one of the body composition assessment components)^4^.

Body composition estimates are based on specific and general mathematical models^5^. Generalist models are more scientifically fragile because they are applicable to different populations and age groups (and therefore have more errors)^6^. Specific models have fewer errors because they have been validated for a specific population^5–7^. The article by Arlindo Sousa et al. provides an example^1^.

The authors^1^ adopted the body density formulas proposed by Petrosky to estimate body fat from skinfolds, as they have been validated in a sample of adults from southern Brazil (i.e., a specific mathematical model)^8,9^, namely: men (18–66 years old): 1.10726863—(0.00081201*X4)+(0.00000212*X4^2^)—(0.00041761*age); women (18–51 years old): 1.02902361—(0.00067159*X4)+(0.00000242*X4^2^)—(0.00026073*age)—(0.00056009*body mass)+(0.00054 649*stature). Finally, the Siri's equation was used to convert body density into body fat percentage^10^.

The above example demonstrates the need to use specific equations for each population and points to the scientific fragility of BIA for body composition assessment, suggesting the question: for which population has BIA been validated? What mathematical models are available in their software? BIAs used in assessment routines do not demonstrate the equations used to estimate body density and body composition, and this is a serious problem due to it being a doubly indirect method for this purpose. In other words, to determine the margin of error in the assessment, it is necessary to know which mathematical model was used, but manufacturers are not clear about this.

Some scientists may suggest reliability and agreement studies^11–14^, but this does not solve the problem of the scientifically fragile nature of BIA for body composition assessment, as positive results may be obtained purely by the role of chance in scientific discovery, since it is not known which population is being specifically studied by the BIA software. In contrast, if the BIA happens to use generalist methods, this increases the chance of obtaining positive results—but generalist methods have long been discouraged^5^.

## Data Availability

The datasets generated and/or analyzed during the current study are available from the corresponding author upon reasonable request.
